# Interest of a simple on-line screening registry for measuring ICU burden related to an influenza pandemic

**DOI:** 10.1186/cc11412

**Published:** 2012-07-09

**Authors:** Jean-Christophe Marie Richard, Tài Pham, Christian Brun-Buisson, Jean Reignier, Alain Mercat, Gaëtan Beduneau, Bernard Régnier, Bruno Mourvillier, Christophe Guitton, Matthias Castanier, Alain Combes, Yves Le Tulzo, Laurent Brochard

**Affiliations:** 1 Service de Réanimation Médicale, Centre Hospitalier Universitaire Charles Nicolle, 1 rue de Germont, Rouen, 76031, France; 2 UPRES EA 3830 (IFR MP23), Institute for Biomedical Research, 22 Boulevard Gambetta, Rouen, 76183, France; 3 Department of Intensive Care, Geneva University Hospital and Geneva University, 4 Rue Gabrielle-Perret-Gentil, Geneva, 1205, Switzerland; 4 Service de Réanimation Médicale, AP-HP, Groupe Hospitalier Universitaire Henri Mondor, 51 Avenue du Maréchal de Lattre de Tassigny, Créteil, 94000, France; 5 Inserm U955, Institut Mondor de Recherche Biomédicale, Université Paris-Est Créteil, 61 Avenue du Général de Gaulle, Créteil, 94010, France; 6 Service de Réanimation Polyvalente, Centre Hospitalier Départemental de la Vendée, Les Oudairies, La Roche sur Yon, 85000, France; 7 Service de Réanimation Médicale et de médecine hyperbare, Centre Hospitalier Universitaire d'Angers, 4 rue Larrey, Angers, 49933, France; 8 LUNAM, Université Nantes Angers Le Mans, 19 bis rue La Nouë Bras de Fer, Nantes, 44200 France; 9 Service de Réanimation Médicale, AP-HP, Centre Hospitalier Universitaire Bichat Claude Bernard, 46, rue Henri-Huchard, Paris, 75018, France; 10 Service de Réanimation Médicale, Centre Hospitalier Universitaire l'Hôtel Dieu, 1 place Alexis-Ricordeau, Nantes, 44093, France; 11 Service de Réanimation, détresses respiratoires et infections sévères, AP-HM, Hôpital Nord, Chemin des Bourelly, Marseille, 13915, France; 12 Service de Réanimation Médicale, AP-HP, Centre Hospitalier Universitaire La Pitié Salpétrière, 47 Boulevard de l'Hôpital, Paris, 75013, France; 13 Service de Maladies Infectieuses et Réanimation médicale, Centre Hospitalier Universitaire Pontchaillou, Rennes, 35033, France; 14 Inserm-0203, Centre d'Investigation Clinique, Université Rennes 1, 2 rue Henri Le Guilloux, Rennes, 35033, France

## Abstract

**Introduction:**

The specific burden imposed on Intensive Care Units (ICUs) during the A/H1N1 influenza 2009 pandemic has been poorly explored. An on-line screening registry allowed a daily report of ICU beds occupancy rate by flu infected patients (Flu-OR) admitted in French ICUs.

**Methods:**

We conducted a prospective inception cohort study with results of an on-line screening registry designed for daily assessment of ICU burden.

**Results:**

Among the 108 centers participating to the French H1N1 research network on mechanical ventilation (REVA) - French Society of Intensive Care (SRLF) registry, 69 ICUs belonging to seven large geographical areas voluntarily participated in a website screening-registry. The aim was to daily assess the ICU beds occupancy rate by influenza-infected and non-infected patients for at least three weeks. Three hundred ninety-one critically ill infected patients were enrolled in the cohort, representing a subset of 35% of the whole French 2009 pandemic cohort; 73% were mechanically ventilated, 13% required extra corporal membrane oxygenation (ECMO) and 22% died. The global Flu-OR in these ICUs was only 7.6%, but it exceeded a predefined 15% critical threshold in 32 ICUs for a total of 103 weeks. Flu-ORs were significantly higher in University than in non-University hospitals. The peak ICU burden was poorly predicted by observations obtained at the level of large geographical areas.

**Conclusions:**

The peak Flu-OR during the pandemic significantly exceeded a 15% critical threshold in almost half of the ICUs, with an uneven distribution with time, geographical areas and between University and non-University hospitals. An on-line assessment of Flu-OR via a simple dedicated registry may contribute to better match resources and needs.

## Introduction

In the fall of 2009, the reported incidence of patients infected with the pandemic influenza A(H1N1) virus in France exceeded the usual incidence of seasonal flu [1,2]. Notification of all patients infected with A(H1N1) virus became mandatory from 1 July to 2 November 2009 but later was restricted to intensive care unit (ICU) admissions because of a large and rapid increase in the number of cases [3].

**Figure 1:**
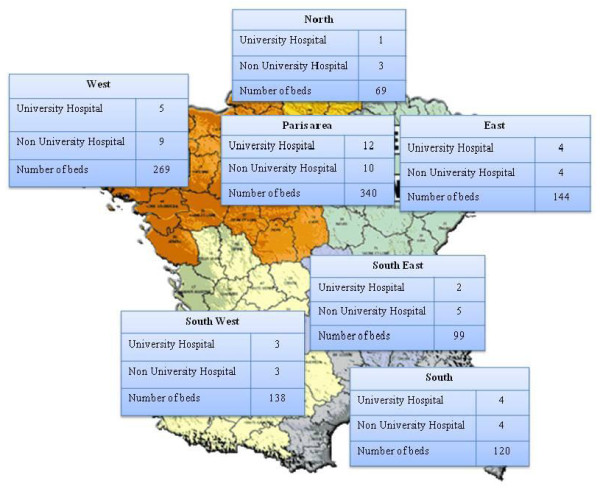
**Number of university and non-university hospitals participating in the study and total number of beds of participating intensive care units in the seven 'defense areas'**.

Taking into account observations made during the early stage of the pandemic in other countries [4-6] (especially in the Southern hemisphere [7-11]), the French Ministry of Health organized the response to the pandemic according to the possible needs of seven regions, so-called 'defense areas', in order to regulate the supply of equipment (Figure 1). The purpose of this plan was to distribute resources equitably across regions while avoiding any shortage in ICU beds. Extracorporal membrane oxygenation (ECMO) devices and ICU ventilators were distributed in the reference centers of each defense area to cover the French territory. A 15% rate of ICU bed occupancy by flu patients in a region was indicated as a critical threshold to consider cancellation of scheduled surgical activities.

At the same time, we recorded data corresponding to the cohort of ICU patients through a large research network on mechanical ventilation (REVA-SRLF registry) [12]. In addition, we designed a dedicated website screening registry to prospectively assess the specific burden related to the A(H1N1) pandemic in ICUs recruited on a voluntary basis. We proposed that a relatively simple screening registry would be able to give a much more exact picture of the respective burden on the different ICUs, geographical areas, and university versus non-university centers. Here, we report the exact rate of ICU bed occupancy by flu-infected patients (Flu-OR) during the pandemic in a representative subset of French ICUs.

## Materials and methods

The French REVA-SRLF registry was a multi-center prospective observational survey based on a website registry, and several results of this registry have been published elsewhere [13-15]. In brief, from November 2009 (week 09-45) through January 2010 (week 10-03), 89 out of 108 ICUs participating in the general registry (representing one fourth of all French ICUs) accepted an invitation to participate in a website screening registry to do a daily assessment of the rate of ICU bed occupancy by influenza-infected and non-infected patients. Sixty-nine ICUs belonging to either referral university hospitals (31 ICUs) or general hospitals (38 ICUs) eventually completed the daily screening for at least three consecutive weeks and admitted at least one A(H1N1)-infected patient and were kept for the present analysis. Twenty ICUs that, for organization reasons or for lack of A(H1N1) patients, did not complete three consecutive weeks were excluded from the analysis.

Recording of patients' data in the registry was approved by the national commission for protection of patients' rights and electronic data recording. The study was approved by the ethics committee of the French Society of Intensive Care (SRLF). Informed consent was waived in agreement with the observational design of the study.

### Cohort study

Suspected infection was proven by means of a polymerase chain reaction eventually completed by serologic analysis. When positive, the patient was considered a 'confirmed case'. A typical clinical flu presentation associated with a negative test was considered a 'suspected case' when no other etiology was found. Suspected as well as confirmed cases were enrolled in the present study. Admission data consisted of dates and times of admission to the hospital and ICU; age; sex; pregnancy status; fatal underlying disease defined within the McCabe [16] classification; the Simplified Acute Physiology Score III (SAPS III) [17], which is a severity score ranging from 0 to 217; immunosuppression and its cause; history of chronic respiratory disease, diabetes, or chronic heart failure; weight and height; and pregnancy. A follow-up during the ICU hospitalization, including time and duration of ventilation, antiviral and corticosteroid use, use of rescue therapy including ECMO, and the cause of death, was also performed.

### Specific screening registry

A specific website registry (screening registry) was designed in order to do a daily assessment of the occupancy rate related to the management of A(H1N1) non-ventilated or ventilated patients.

**Figure 2:**
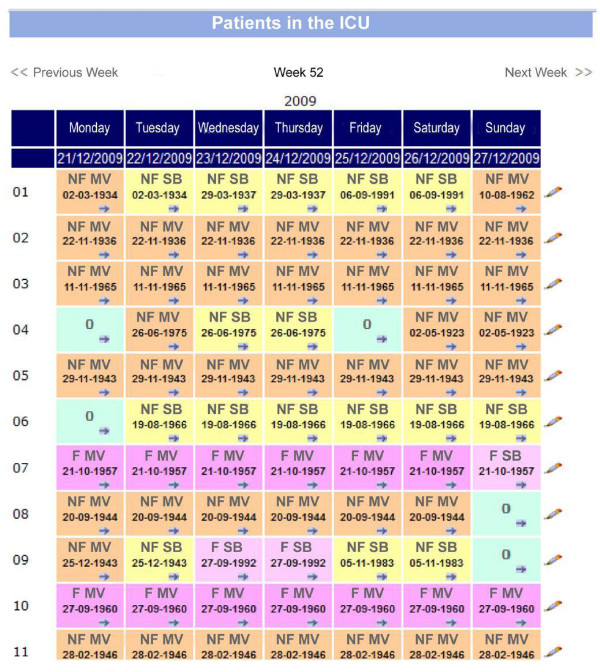
**Original version of the online registry page dedicated to the screening study for an intensive care unit (ICU) of 10 beds**. Each column represents a day of the week, and each line represents a bed of this ICU. Each box of this table gives the birth date and status of the patient occupying this particular bed on this particular day. F, flu; MV, mechanical ventilation; NF, non-flu; SB, spontaneous breathing; 0, unoccupied bed.

Isn't 'in order to daily assess the occupancy rate...' better? Baseline information on the number of available beds, ventilators, and staffing was collected. For each participating unit, a customized table representing the number of available beds on each day of the week was displayed on the website (Figure 2). Each available bed was characterized by using a specific code on a daily basis to identify whether it was occupied or used by a non-infected patient or a flu-infected patient; the ventilation status (mechanically ventilated or not) of each patient was also coded. The number of available beds could be modified each day in case of the closing or opening of additional beds. Online completion of the registry required less than 30 minutes per day for a 20-bed ICU and usually was performed every morning after checking the patients' status during the first round. To facilitate postponed data recording, a table corresponding to the week in progress was available on the website for printing. The website was designed by a professional who paid great attention to the user-friendliness of the interface. Each participating center received a weekly electronic update to provide information on the evolution of the pandemic and encourage completion of the registry. When needed, reminders were automatically displayed on the website.

Based on these data, the ICU bed Flu-OR was computed as the number of bed-days per week occupied by flu patients divided by the total number of bed-days occupied per week and was expressed as a percentage. A weekly Flu-OR was calculated for each participating ICU and for each defense area in the course of the pandemic, differentiating ventilated from non-ventilated patients. We also differentiated Flu-OR observed in university hospitals from that in non-university hospitals.

### Data collection and quality control assessment

Data collected were directly downloaded as electronic .xls files from the REVA web registry. The data management and the analysis were performed by CB-B, TP, and J-CMR. The database was completed when needed after direct contact with the ICU physicians. Duplicate notifications were systematically checked, and patients transferred from one participating ICU to another were counted as a single admission.

### Statistical analysis

Descriptive statistics included frequency analysis - percentages and corresponding 95% confidence intervals (CIs) - for categorical variables and means and standard deviations or medians and interquartile ranges (IQRs) for continuous variables. Differences in medical and demographic characteristics according to outcomes or in Flu-OR between types of hospitals were tested by using a chi-squared test for categorical variables and a Student *t *test for continuous parametric variables. All statistical tests were two-sided, and *P *values of 0.05 or less denoted statistical significance. Statistical analysis was performed with R software packages [18].

## Results

### Cohort study

Three hundred ninety-one patients with A(H1N1) were admitted from 26 September 2009 to 10 February 2010 in the 69 ICUs participating in the screening registry and were included in this study. Among them, 349 (89%) had a confirmed influenza A infection. This subset of 391 patients represents 36.7% of the whole cohort of French influenza-infected adult ICU patients [3]; they had the same overall characteristics, except for a higher rate of immunosuppression (Table 1). Tables 2 and 3 show the baseline characteristics and main risk factors for flu recorded in these patients according to survival or to the intensity of ventilatory support. Mechanical ventilation was provided to 286 (73%) patients, 231 (59%) fulfilled criteria for acute respiratory distress syndrome, and 50 (13%) required additional ECMO. The mortality rate of the whole cohort was 22%.

**Table 1:** Characteristics of the patients reported to the national health surveillance system [3] and the subgroup included in the screening study

Baseline characteristics	Screening cohort (*n *= 391)	NIPHS (*n *= 1,065)
Age in years	46 (33-57)	49 (35-58)
Male	210 (53%)	562 (53%)
No risk factor of complication	87 (22%)	191 (18%)
Immunosuppression	89 (23%)	82 (8%)
Asthma	46 (12%)	135 (13%)
Chronic obstructive pulmonary disease	77 (20%)	214 (20%)
Chronic heart failure	33 (8%)	84 (8%)
Diabetes	53 (13%)	125 (12%)
Obesity: body mass index > 30	115 (29%)	84 (27%)
Pregnancy	18 (4.6%)	58 (6%)
Death	87 (22.2%)	217 (20.4%)

Except for 'Age in years', which is expressed as mean (interquartile range), values are expressed as number (percentage). NIPHS, National Institute for Public Health Surveillance.

**Table 2:** Baseline characteristics and comparison of survivors with the deceased

Baseline characteristics	Survivors (*n *= 304)	Deceased (*n *= 87)	*P *value
Age in years	45 (16)	50 (17)	0.01
Male	159 (52%)	51 (59%)	0.35
McCabe 1	241 (79%)	50 (57%)	< 10^-4^
McCabe 2 or 3	63 (21%)	37 (42%)	< 10^-4^
No risk factor of complication	68 (22%)	19 (22%)	0.97
Immunosuppression	51 (17%)	38 (44%)	< 10^-4^
Asthma	46 (15%)	0 (0%)	< 10^-4^
Chronic obstructive pulmonary disease	61 (20%)	16 (18%)	0.84
Chronic heart failure	26 (9%)	7 (8%)	0.94
Diabetes	40 (13%)	13 (15%)	0.80
Obesity: body mass index > 30	86 (28%)	29 (33%)	0.43
Pregnancy	17 (6%)	1 (1%)	0.14

Except for 'Age in years', which is expressed as mean (standard deviation), and *P *values, values are expressed as number (percentage). McCabe 1 = no or non-fatal underlying disease; McCabe 2 = ultimately fatal disease (< 5 years); McCabe 3 = rapidly fatal disease (< 1 year).

**Table 3:** Baseline characteristics and clinical course according to intensity of ventilatory support

Baseline characteristics	Total	No mechanical ventilation	Mechanical ventilation without ARDS	ARDS without ECMO	ECMO
Number of patients (percentage)	391	105 (27%)	55 (14%)	181 (46%)	50 (13%)
Age in years, mean (SD)	46 (16)	40 (16)	52 (18)	48 (15)	37 (12)
SAPS III, mean (SD)	52 (17)	41 (13)	53 (14)	56.5 (17)	58.3 (17)
Duration of ventilation in days, median (IQR)	12 (5-24)	0	2 (4-10)	13 (7-21)	26 (12-37)
Length of stay in ICU in days, median (IQR)	10 (5-24)	4 (3-6)	8 (5-16)	17 (9-27)	26 (14-42)
Obesity, number (percentage)	115 (29%)	20 (19%)	16 (21%)	60 (33%)	23 (46%)
Mortality, number (percentage)	87 (22.2%)	2 (1.9%)	7 (12%)	56 (30.1%)	22 (44%)

ARDS, acute respiratory distress syndrome; ECMO, extracorporal membrane oxygenation; ICU, intensive care unit; IQR, interquartile range; SAPS III, Simplified Acute Physiology Score III; SD, standard deviation.

### Screening registry

Figure 1 shows the distribution of the 69 ICUs participating in the screening registry across the seven defense areas as well as the distribution of ICU beds belonging to university and non-university hospitals. The median number of beds per ICU (including intermediate-care beds) was 15 (IQR of 12 to 21), representing a total of 942 ICU beds and 220 intermediate-care beds.

**Figure 3:**
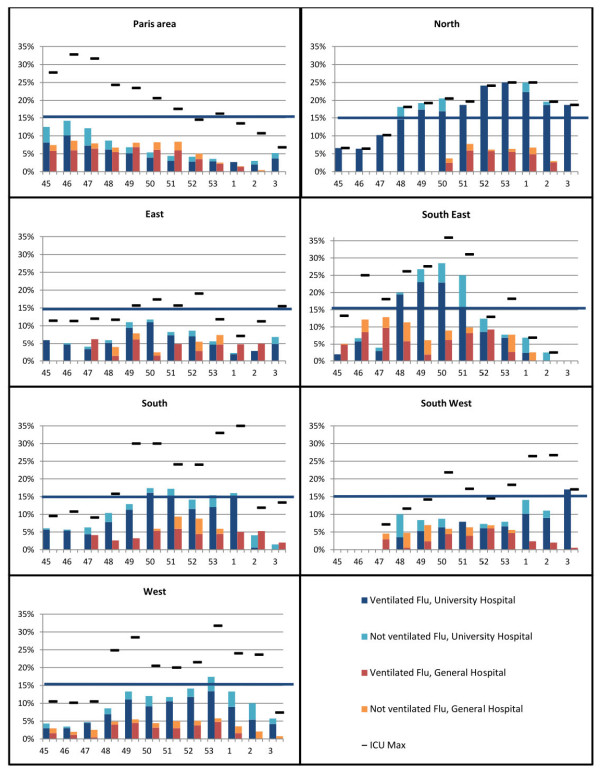
**Rate of intensive care unit (ICU) bed occupancy by flu-infected patients in the seven 'defense areas'**. For each defense area, the table shows the weekly occupancy rate (from week 45 of 2009 to week 3 of 2010) according to the type of ICU (university or non-university) and the rate of ventilated and non-ventilated patients. The black marks indicate the maximal occupancy rate of an ICU of this area.

Figure 3 shows the weekly and maximal Flu-OR in the seven defense areas. The peak of the pandemics varied across the different geographical defense areas as illustrated by the profiles of the mean Flu-OR in each region (Figure 3). The maximal Flu-OR observed in any ICU in each region is also indicated. A Flu-OR of above 15% was recorded in 32 individual ICUs, for a total of 103 weeks out of the accumulated 623 weeks of screening across the 69 ICUs: 16.5% of the screening weeks (95% CI of 13.6% to 19.4%). The percentage of screened weeks with Flu-OR of above 15% varied between defense areas from 8.5% (east) to 34.3% (north).

**Figure 4:**
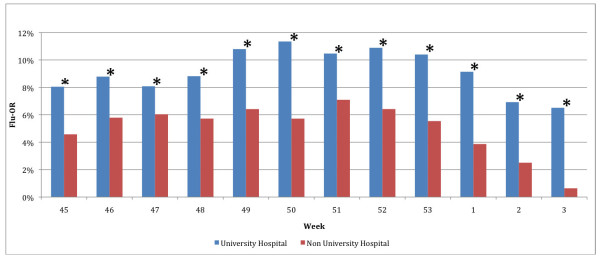
**Comparison of rates of bed occupancy by flu-infected patients (Flu-OR) in university and non-university hospitals after all seven 'defense areas' were pooled together**. For each week of the pandemic (from week 45 of 2009 to week 3 of 2010), national Flu-OR of university intensive care units (ICUs) (blue columns) was higher than that of non-university ICUs (red columns). *P < 0.05.

The pooled Flu-OR calculated over the whole study period (including university and non-university hospital) for the entire country was 7.6% and varied across the seven defense areas from 6.1% (east) to 10.5% (southeast). At each week, this rate was significantly higher in university hospitals than in non-university hospitals (Figure 4). During the pandemic period, none of the participating hospitals used the threshold level of 15% ICU bed occupancy rate to modify hospital admission policy or bed availability.

## Discussion

In this prospective observational study using a dedicated online screening registry designed to assess the daily burden of the A(H1N1) influenza pandemic on French ICUs, we found important variations in the actual influenza burden between geographical areas, university and non-university hospitals, and time. In several individual ICUs, our screening registry has permitted us to observe peak occupancy rates greatly exceeding those calculated by averaging data observed in the largest defense areas. Our findings suggest that the organization of future pandemic response plans can greatly benefit from online data obtained in almost real time [19,20]. A dedicated online registry able to assess the week-by-week Flu-OR in each ICU may help to better distribute resources according to the actual needs. Even if ICUs were encouraged to do a daily assessment of the presence of patients with A(H1N1), we chose to report the calculation per week first to be consistent with the French organization and the National Institute for Public Health Surveillance (NIPHS), which displayed the time course of the pandemic weekly, and also to simplify data notification for participating centers. In fact, in collaboration with the NIPHS, we developed a strong communication strategy via the REVA website to simplify and therefore encourage rigorous notification.

Flu-ORs calculated per week in the present study were comparable to and often higher than those observed in the Southern hemisphere. Over the 3-month pandemic period in Australia and New Zealand [11], the ANZICS (Australian and New Zealand Intensive Care Society) investigators reported that an average of 5.2% of available ICU bed-days were occupied by patients with H1N1 infection, whereas the peak percentage ranged from 8.9% to 19% [11]. In our study, 7.6% of the beds of enrolled ICUs were occupied by influenza-infected patients during the whole study period, and a maximum Flu-OR recorded per week in individual ICUs reached 35%. Inclusion of the 10 ICUs that had not admitted any patients with A(H1N1) would have lowered global Flu-OR but would not have changed the maximal Flu-ORs that we observed. The ICU length of stay, however, may have impacted the Flu-OR since a large difference between the two reports was observed: the median durations of ICU stay were 7.4 days (IQR of 3 to 16) in the ANZICS study and 10 days (IQR of 5 to 24) in French ICUs. Uneven time and regional distribution may also impact the Flu-OR calculations.

In the ANZICS investigation, the number of days that ICU beds were occupied by infected patients per region was calculated by multiplying the total number of patients by their length of stay. This approach allowed us to estimate a peak Flu-OR per region but not per individual ICU. The results of our registry show that this can greatly underestimate true peak activities in some ICUs. This is of particular importance since H1N1 burden changed rapidly over time and from one ICU to the other. The web-based screening registry specifically developed for our study allowed a daily assessment of the ICU occupancy rate in individual ICUs with an accuracy similar to that in larger regions. For example, observations reported from the Paris area (Figure 3) showed that the maximal Flu-OR significantly differed from the weekly Flu-OR calculated within all ICUs belonging to that region. Overall, our observations suggest that an accurate estimation of the influenza burden on ICUs requires a daily and real-time Flu-OR assessment, which seems feasible in view of our findings.

In anticipation of the flu surge, health authorities decided that, in order to alleviate the pressure on ICU beds, planned surgery activity should be cancelled when Flu-OR exceeded 15%. We contacted each participating ICU and confirmed that no canceling of scheduled surgery had been decided although Flu-OR exceeded 15% in many ICUs. Such peak activities occurred for more than 103 weeks among 32 units. Future analysis may help to refine this threshold and thus may have to take into account how long it is exceeded. The magnitude of a pandemic and its consequences on the organization of a health-care system depend on several parameters and are notoriously difficult to predict [21,22]. Therefore, experiences reported during the first waves throughout the world were useful to better elaborate forecasts taking into account different attack rates and epidemic wave durations [23,24]. Data gathered in Canada during the first wave were applied to a variety of second-wave models to determine its impact on ICU and ventilator demand [21,25]. An attack rate of greater than 20% can result in significant shortages in ICU beds and ventilators [21]. Retrospective estimations showed that attack rates in Europe [26], North America, and Australia did not exceed 10% during each of the 2009 pandemic waves [27]. The overall impact also greatly depends on the durations of mechanical ventilation and ICU stay. High attack rate combined with a short epidemic duration and long expected duration of mechanical ventilation represents the worst scenario in terms of bed occupancy rate and thus the maximal burden. Given a 20% attack rate with a similar clinical presentation, French ICU resources in university hospitals would probably have been overwhelmed, according to our observations.

A weakness of our study is that only a subset of French ICUs participated in the screening. Our cohort represented 35% of the whole French cohort reported during the same period to the health ministry. The distribution of the subset of university and non-university hospitals participating in this study within the seven areas (except in the northern area) suggests that French ICU health-care resources were reasonably well represented (Figure 1). This is supported by the comparability of baseline characteristics of patients within the present cohort to those of the entire French cohort (Table 1) [3].

General inferences that can be made from this study are limited because of the difficulties in extrapolating from one health-care system to another. But regardless of the system, the primary goal here was to be able to assess, as closely to reality as possible, the related burden of an epidemic, and such an assessment was made possible by the specific screening registry.

## Conclusions

Revisiting the pandemic plans in light of emergent findings is certainly a key issue to be able to cope with further pandemic waves. Our observations suggest that the specific activity related to critically ill H1N1-infected patients varied widely according to time, regions, and individual ICUs as well as within larger areas. We report maximal ICU Flu-ORs that significantly exceed the 15% predefined critical threshold. The website registry described here and tested during the first pandemic wave in France allowed a real-time awareness of bed utilization and capacity.

## Key messages

• A simple online registry permits us to accurately describe specific H1N1 ICU burden in real time.

• ICU burden related to H1N1-infected patients varied widely according to time, regions, and individual ICUs.

• In most ICUs, the maximal rate of bed occupancy by patients with flu exceeded the predefined 15% critical threshold.

• Online assessment of Flu-OR in the ICU may help to better match resources and needs in case of new H1N1 pandemics.

## Abbreviations

ANZICS: Australian and New Zealand Intensive Care Society; CI: confidence interval; ECMO: extracorporal membrane oxygenation; Flu-OR: rate of bed occupancy by flu-infected patients (in the intensive care unit); ICU: intensive care unit; IQR: interquartile range; NIPHS: National Institute for Public Health Surveillance; REVA: Recherche en Ventilation Artificielle (Research on Mechanical Ventilation); SRLF: Sociéte de Réanimation de Langue Française (French Society of Intensive Care).

## Competing interests

The authors declare that they have no competing interests.

## Authors' contributions

J-CMR helped to conceive of the study and participated in its design and coordination, helped to design the website registry and to coordinate data management, and participated in the editing of the manuscript. TP helped to conceive of the study and participated in its design and coordination, helped to coordinate data management, and participated in the editing of the manuscript. CB-B helped to conceive of the study and participated in its design and coordination and helped to design the website registry and to coordinate data management. AM helped to conceive of the study and participated in its design and coordination and helped to design the website registry. LB helped to conceive of the study and participated in its design and coordination, helped to design the website registry, and participated in the editing of the manuscript. GB helped to design the website registry and actively participated in the study as a REVA correspondent from the six most active centers. JR and BR (president and member of the SRLF, respectively) helped and encouraged participating centers to complete the screening registry. BM, CG, MC, AC, and YLT actively participated in the study as REVA correspondents from the six most active centers. All authors read and approved the final manuscript.
